# Reactive Nodular Fibrous Pseudotumor: Case Report and Review of the Literature

**DOI:** 10.1155/2014/421234

**Published:** 2014-03-30

**Authors:** Rawand Salihi, Philippe Moerman, Dirk Timmerman, Dominique Van Schoubroeck, Katya Op de beeck, Ignace Vergote

**Affiliations:** ^1^Division of Gynaecological Oncology, Department of Obstetrics & Gynaecology, Leuven Cancer Institute, University Hospital Leuven, Herestraat 49, 3000 Leuven, Belgium; ^2^Division of Pathology, Leuven Cancer Institute, University Hospital Leuven, Herestraat 49, 3000 Leuven, Belgium; ^3^Screening, Diagnostics and Biomarkers, Leuven Cancer Institute, University Hospital Leuven, Herestraat 49, 3000 Leuven, Belgium

## Abstract

We will describe a case of a patient diagnosed with a rare identity of a benign lesion, “reactive nodular fibrous pseudotumor” (RNFP). It is a tumor which preoperatively can present as a malignant tumor and is only reported in 19 cases. According to the very limited amount of information on this tumor in the literature it is mostly seen after trauma or intraperitoneal inflammation. Our case is the second one of RNFP associated with endometriosis, which is a frequently seen intraperitoneal inflammation process in women. Knowledge that these large pseudotumoral lesions can occur is important to direct the management of these patients.

## 1. Introduction

A multitude of tumors can occur in the peritoneal cavity. Correct diagnosis is of paramount importance for proper treatment. We recently observed a patient with an intra-abdominal mass, diagnosed histopathologically as “reactive nodular fibrous pseudotumor” (RNFP). This is a benign lesion, often mimicking a malignant tumor preoperatively. The pathogenesis might be related to intraperitoneal “trauma” such as endometriosis. It is previously reported in only 19 cases.

## 2. A Case Report

**Figure 1:**
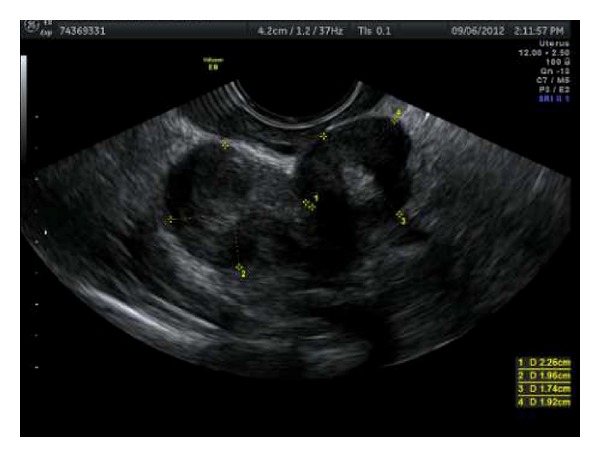
Ultrasound: multiple solid masses in the pouch of Douglas (23 mm × 20 mm and 17 mm × 19 mm), with myometrial aspect and central echogenic parts. They are attached to the left ovary and rectosigmoid but without invasion of its muscular wall.

**Figure 2:**
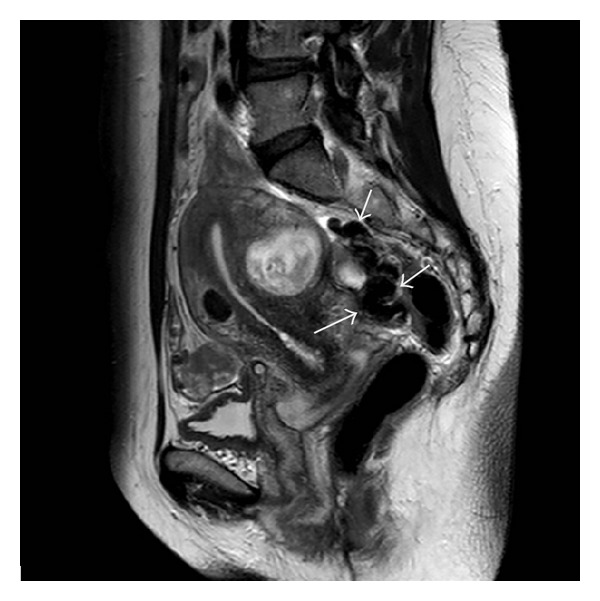
Sagittal T2-weighted MR image shows polylobular masses in the pouch of Douglas with very low signal intensity.

**Figure 3:**
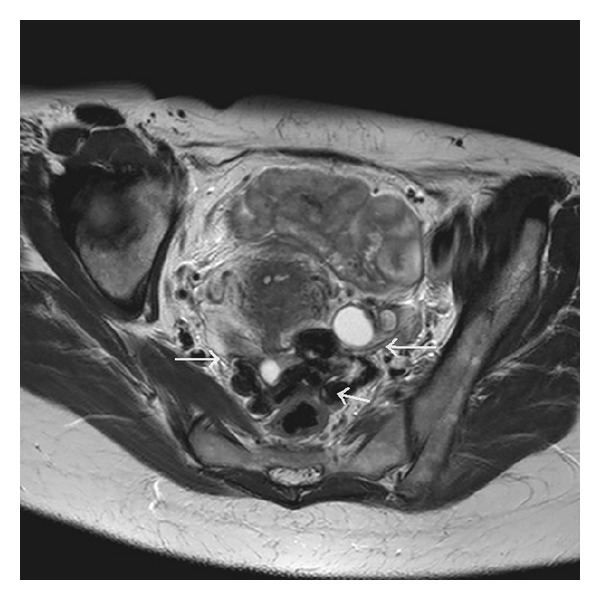
Axial T2-weighted MR image shows polylobular masses in the pouch of Douglas with very low signal intensity.

**Figure 4:**
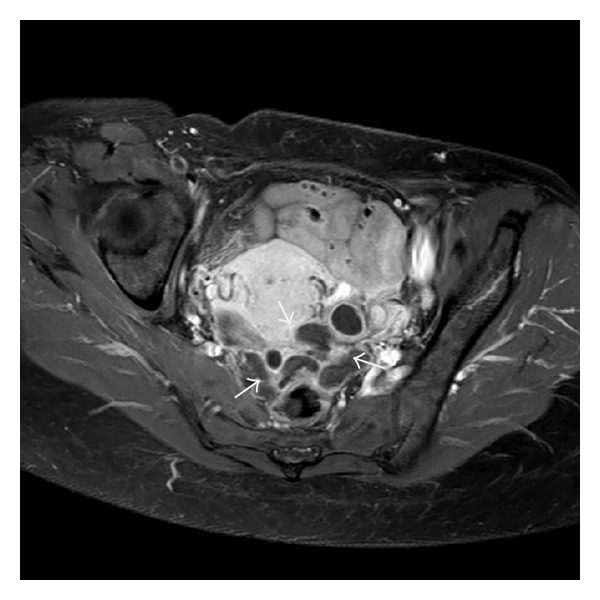
On axial fat-suppressed T1-weighted images after gadolinium administration the masses are strongly hypovascular with a peripheral rim-like enhancement.

**Figure 5:**
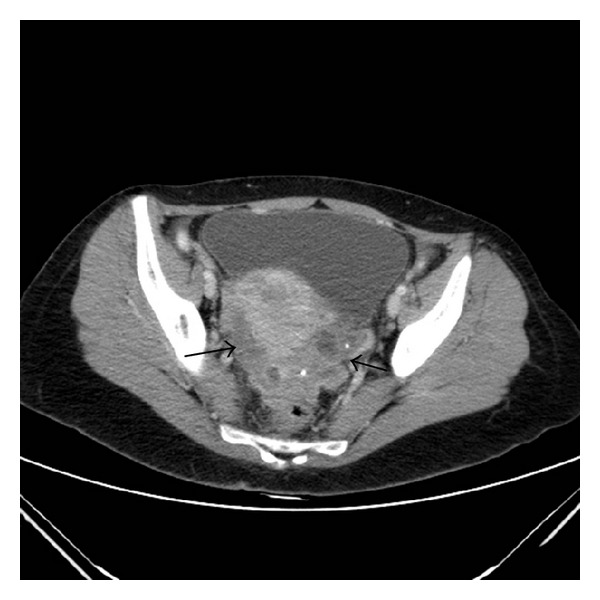
Axial CT image after contrast administration shows heterogeneous iso- to hypoattenuating masses in the pouch of Douglas. Inside we can see some punctiform calcifications.

A 45-year-old woman was hospitalized in another hospital, because of intractable menometrorrhagia, pain, and gradual abdominal swelling. A vaginal hysterectomy was planned and performed but shortly had to be stopped because of bleeding. During the procedure intraperitoneal lesions were seen and biopsied. Pathology shows no signs of malignancy. Afterwards the patient was sent to our hospital for further diagnosis. Her medical history mentioned no other abdominal surgery, migraine headaches, or other major incidents. Her obstetrical history recorded an uncomplicated vaginal delivery. Clinical examination showed no particularities. Biochemically we found no abnormalities and serum CA125 levels were normal. Gynecological ultrasound (Figure 1) demonstrated not only diffuse uterine adenomyosis and myomas, but also multiple solid masses in the Douglas pouch, attached to the left ovary and rectosigmoid but without invasion of its muscular wall. MRI (Figures 2, 3, and 4) and CT (Figure 5) confirmed the presence of solid and strongly hypovascular masses in the pouch of Douglas. A similar smaller lesion was present at the caudal border of the transverse colon. The very low signal intensity on T1- and T2-weighted MR images was very suggestive for fibrotic tissue. On imaging the diagnosis of disseminated intraperitoneal leiomyomatosis was suggested, but other fibrous tumoral lesions or malignancy could not be excluded.

Because of the age of the patient and because a malignant disease could not be excluded, it was decided to perform a total abdominal hysterectomy with bilateral salpingo-oophorectomy. Laparotomic peritoneal exploration showed normal female genital organs, but multiple fibrous plaques and nodules on the sigmoid. A sigmoid resection with end-to-end anastomosis was performed as the tumor was widespread on the sigmoid involving the vasculature. Frozen section analysis reported a benign lesion with low cellularity, chronic inflammation, and no atypia. Postoperative recovery was uneventful and our patient could leave the hospital within a week. Until now there are no signs of recurrence.

## 3. Pathological Examination

**Figure 6:**
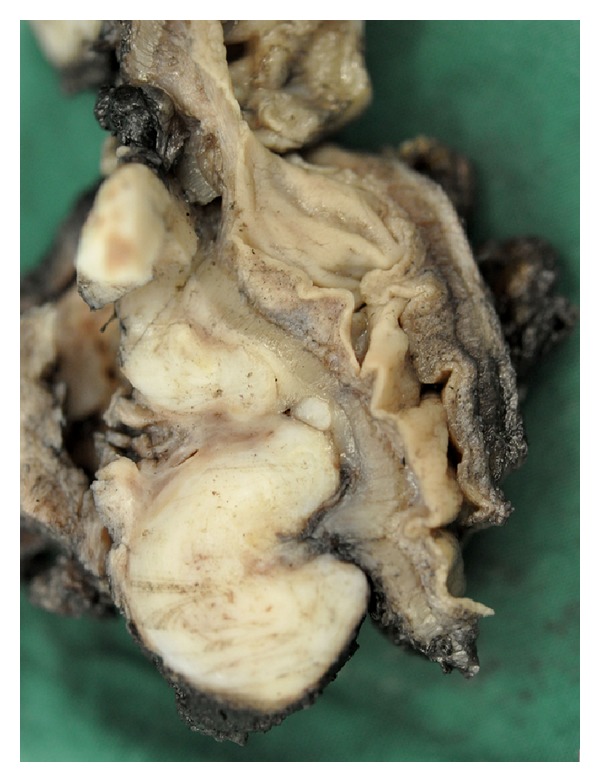
Several confluent well-circumscribed fibrotic nodules in the pouch of Douglas. Note the intact muscular wall and mucosa of the sigmoid.

**Figure 7:**
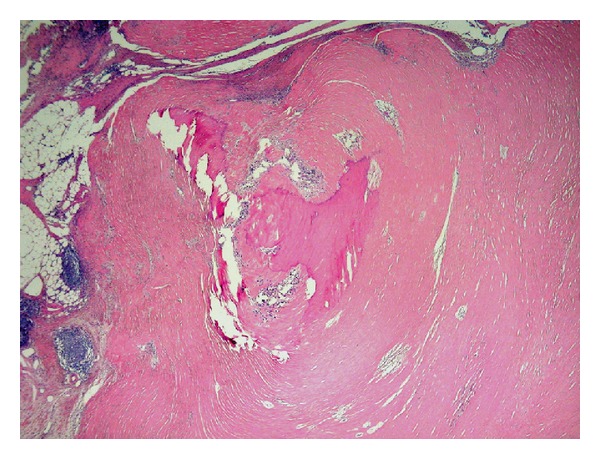
Histology of a fibrotic nodule (H&E stain, low magnification). It consists of concentric layers of paucicellular hyalinized collagen, with central dystrophic calcification. The surrounding fat tissue contains foci of lymphocytic inflammation.

**Figure 8:**
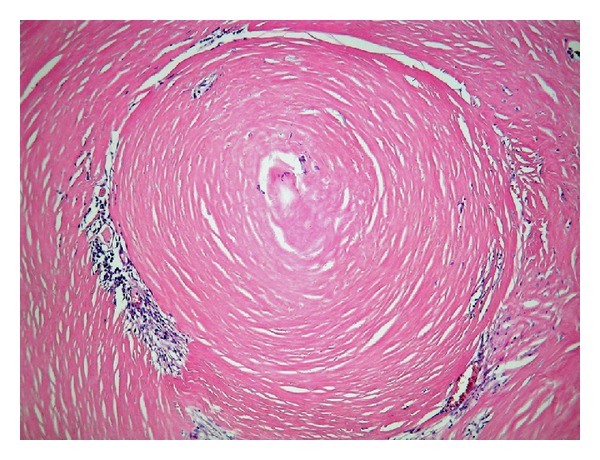
Histology of RNFP (H&E stain, high magnification).

The surgical specimen consisted of the uterus with both adnexa, a sigmoid segment, and the omentum. Gross examination revealed white, hard nodules with a smooth surface on the omentum, sigmoid, and right ovary. The largest nodule measured 7 × 6.3 × 3 cm and was located on the sigmoid. Histologically, there were multiple fibrocollagenous nodules on the omentum, pelvic wall, right ovary, sigmoid, and Douglas pouch (Figure 6). These nodules consisted of concentric layers of paucicellular hyalinized collagen, often with central dystrophic calcification (Figures 7 and 8). At the edges there was chronic inflammation and fibroblastic proliferation. Cytological atypia and mitoses were absent. Some nodules were closely associated with endometriosis.

## 4. Discussion

Pathologically, the resected lesions are consistent with the so-called “reactive nodular fibrous pseudotumor” (RNFP), first reported by Yantiss et al. [1] in 2003. It was described as poorly formed fascicles and aggregates of fibroblasts and myofibroblasts, admixed with a sparse lymphocytic inflammatory infiltrate enmeshed within a densely collagenous stroma. Typically these tumors stain immunohistochemically with fibroblastic and myofibroblastic markers.

Since 2003, a total of 19 cases of this entity have been recognized. It is considered as a nonneoplastic (myo)fibroblastic proliferation, representing an inappropriate postinflammatory response to injury. All kinds of injury have been proposed: abdominal surgery (5x), peptic ulcers (2x), foreign body ingestion (2x), perforated duodenal diverticulitis, endometriosis in combination with the use of ergotamine, and chronic bowel obstruction. In our case we can only recognize endometriosis as a risk factor.

All previously reported cases are from developed countries (Czech Republic, 8; USA, 6; France, 2; Australia, 1; Turkey, 1; Italy, 1). Most patients are adults above 18 years; one case was a 1-year-old child. There is a clear male-to-female preponderance of 14 to 5. Cases with both solitary (11) and multiple (8) masses have been described [2].

The fact that RNFP often presents with multiple intra-abdominal masses evidently causes clinical concern for malignancy [3]. RNFP has to be differentiated from intra-abdominal inflammatory myofibroblastic tumors and inflammatory fibrosarcoma. The latter two are common in young children and early adulthood and were previously classified into a single category of “inflammatory pseudotumors.” However it is now realized that these lesions have a high propensity for local recurrence and may uncommonly metastasize. For RNFP, until now there are no cases of recurrence.

Another neoplastic entity to consider in the differential diagnosis is a GIST, which commonly has a more brown, fleshy, more variable gross appearance with hemorrhage and necrosis. Histologically these tumors show a more organoid architecture with cellular fascicles of plump spindle cells with eosinophilic cytoplasm separated by delicate fibrous septa and a hyalinized or edematous stroma [4–6].

In the differential diagnosis of RNFP, a number of nonneoplastic lesions have to be considered, for example, the calcified fibrous pseudotumor which shares many features with RNFP. Like RNFP, it frequently occurs intra-abdominally and is a benign soft tissue lesion consisting of a hypocellular spindle cell proliferation within dense collagen. It is accompanied by dystrophic calcifications but is more cellular and contains a mixed inflammatory infiltrate [7]. Sclerosing mesenteritis and retroperitoneal fibrosis are reactive lesions that occasionally occur together. Sclerosing mesenteritis causes diffuse fibrous thickening of the peritoneum. It is caused by chronic irritation and is typically seen in patients with cirrhosis, ascites, peritoneal dialysis, peritoneovenous shunt, endometriosis, or familial Mediterranean fever. It also occurs in association with luteinized thecomas [8, 9]. Retroperitoneal fibrosis may be associated with Riedel's thyroiditis, as well as the use of specific medications including methysergide [10–12]. Nodular fasciitis or nodular fasciitis-like proliferations may also be considered in the differential diagnosis. These lesions are characterized by a highly cellular, mitotically active proliferation of fibroblasts and myofibroblasts in a richly vascularized loose stroma. Finally, intra-abdominal fibromatosis is a solitary, ill-defined, gray-tanned mass with irregular and infiltrative borders, whereas RNFP is usually well-circumscribed.

This is the second case of RNFP with associated endometriosis [13]. The other case with endometriosis was associated with ergotamine use, which was not documented in our case. Endometriosis is a known cause of intra-abdominal inflammation and fibrosis. Knowledge that endometriosis can give rise to such large pseudotumoral lesions is important to direct the management of these patients.

## References

[1] Yantiss RK, Nielsen GP, Lauwers GY, Rosenberg AE (2003). Reactive nodular fibrous pseudotumor of the gastrointestinal tract and mesentery: a clinicopathologic study of five cases. *The American Journal of Surgical Pathology*.

[2] Virgilio E, Pucci E, Pilozzi E, Mongelli S, Cavallini M, Ferri M (2012). Reactive nodular fibrous pseudotumor of the gastrointestinal tract and mesentery giving multiple hepatic deposits and associated with colon cancer. *The American Surgeon*.

[3] Daum O, Vanecek T, Sima R (2004). Reactive nodular fibrous pseudotumors of the gastrointestinal tract: report of 8 cases. *International Journal of Surgical Pathology*.

[4] Berman J, O'Leary TJ (2001). Gastrointestinal stromal tumor workshop. *Human Pathology*.

[5] Brainard JA, Goldblum JR (1997). Stromal tumors of the jejunum and ileum: a clinicopathologic study of 39 cases. *The American Journal of Surgical Pathology*.

[6] Miettinen M, Lasota J (2001). Gastrointestinal stromal tumors—definition, clinical, histological, immunohistochemical, and molecular genetic features and differential diagnosis. *Virchows Archiv*.

[7] Nascimento AF, Ruiz R, Hornick JL, Fletcher CDM (2002). Calcifying fibrous “pseudotumor”: clinicopathologic study of 15 cases and analysis of its relationship to inflammatory myofibroblastic tumor. *International Journal of Surgical Pathology*.

[8] Clement PB, Kurman RJ (2002). Disease of the peritoneum. *Blaustein’s Pathology of the Female Genital Tract*.

[9] Clement PB, Young RH, Hanna W, Scully RE (1994). Sclerosing peritonitis associated with luteinized thecomas of the ovary: a clinicopathological analysis of six cases. *The American Journal of Surgical Pathology*.

[10] Comings DE, Skubi KB, van Eyes J, Motulsky AG (1967). Familial multifocal fibrosclerosis: findings suggesting that retroperitoneal fibrosis, mediastinal fibrosis, sclerosing cholangitis, Riedel’s thyroiditis, and pseudotumor of the orbit may be different manifestations of a single disease. *Annals of Internal Medicine*.

[11] Graham JR, Suby HI, LeCompte PR, Sadowsky NL (1966). Fibrotic disorders associated with methysergide therapy for headache. *The New England Journal of Medicine*.

[12] Hawk WA, Hazard JB (1959). Sclerosing retroperitonitis and sclerosing mediastinitis. *The American Journal of Clinical Pathology*.

[13] Saglam EA, Usubütün A, Kart C, Ayhan A, Küçükali T (2005). Reactive nodular fibrous pseudotumor involving the pelvic and abdominal cavity: a case report and review of literature. *Virchows Archiv*.

